# Lessons of 10 years experience on CCHF in Iran

**Published:** 2011-01-10

**Authors:** S Chinikar, SM Ghiasi, M Moradi, MM Goya, MR Shirzadi, M Zeinali, A Fayaz

**Affiliations:** 1 Arboviruses and Viral Hemorrhagic Fever (National Reference Lab), Pasteur Institute of Iran, Tehran, Iran; 2 Center for Disease Control (CDC), Ministry of Health (MOH), Tehran, Iran

## Background

Crimean-Congo Hemorrhagic Fever (CCHF) is a viral zoonotic disease with high mortality rate in humans caused by CCHF virus (CCHFV) belonging to the genus *Nairovirus,* family *Bunyaviridae*, and congaing a three segment single-stranded RNA genome. The CCHFV is transmitted to humans by bite of infected ticks, by direct contact with blood or tissues of infected livestock and nosocomially. After Chaharmahal-va-Bakhtiari outbreak in 1999 whose serum samples was sent to South Africa for diagnosis, Arboviruses and Viral Hemorrhagic Fevers Laboratory (As National Reference Lab) was established in 2000 to precise and on time laboratory diagnosis of CCHF in the country. The Lab along with CDC of Iran (national health regulator) and Veterinary organization (control program of tick populations and livestock monitoring) are members of National Expert Committee on Viral Hemorrhagic Fevers (NECVHFs) for surveillance and control of CCHF in Iran.

## Methods

Since the establishment of the laboratory as National Ref. Lab, probable human sera, suspected livestock sera and tick samples were analyzed by serological (IgM and IgG ELISA) and molecular (Real-Time and Gel-Based RT-PCR) assays.

## Results

As our result show, the mortality rate of CCHF in the country has been declined in the recent years compared with the early year. Regarding transmission route, most important route of transmission has been through close contact with blood and tissue of infected livestock, so the highest proportion of CCHF infection has been seen in high risk professions such as slaughterers, butchers, farmers. By considering geographical distribution, Sistan-va-Baluchistan not only ranked the most infected province in Iran, but also, the infection has been seen in all years. On the other hand, other most infected provinces included Isfahan, Fars, Khorasan and Yazd respectively, although CCHF infection has been seen in 23 out of 30 provinces of the country. Concerning sex distribution, males are infected with triple times as much as females. Also, in age distribution the disease has been prevalent in age range of 21-40 years old. The CCHFV genome was detected in tick populations collected from different high risk areas. With respecting genetic analysis, Our phylogenetic studies on CCHFV genome extracted from human and tick demonstrated that the Iranian strain have had close relationship with Pakistani (Matin) strain and the genome extracted from both hosts were very similar altogether.

## Conclusion

With efficient trainings for high risk professions, the mortality rates can be decreased in high risk areas. In addition, control and prevention programs such as tick population control may address the decline of the CCHF disease in endemic regions and to block its prevalence to other regions of the country. Lastly, import of livestock to the country should be monitored and population of livestock and tick in high risk areas and endemic regions should be always surveyed with serological and molecular epidemiology. With different data on mortality rate in different endemic regions, more pathogenesis and phylogenetic analysis should be performed.

